# Secondary Metabolites in the Green Synthesis of Metallic Nanoparticles

**DOI:** 10.3390/ma11060940

**Published:** 2018-06-03

**Authors:** Gregory Marslin, Karthik Siram, Qaisar Maqbool, Rajendran Kamalabai Selvakesavan, Dariusz Kruszka, Piotr Kachlicki, Gregory Franklin

**Affiliations:** 1 Ratnam Institute of Pharmacy and Research, Nellore 524346, India; marslingregory@gmail.com; 2 Department of Pharmaceutics, PSG College of Pharmacy, Coimbatore 641004, India; karthiksiram@gmail.com; 3 Institute of Plant Genetics of the Polish Academy of Sciences, Poznan 60479, Poland; Qaisar.vu@gmail.com (Q.M.); kesavanrks@gmail.com (R.K.S.); dmkruszka@gmail.com (D.K.); pkac@igr.poznan.pl (P.K.); fgre@igr.poznan.pl (G.F.)

**Keywords:** nanoparticles, green synthesis, secondary metabolites, flavonoids, bioactivities, nano-harvesting, drug discovery

## Abstract

The ability of organisms and organic compounds to reduce metal ions and stabilize them into nanoparticles (NPs) forms the basis of green synthesis. To date, synthesis of NPs from various metal ions using a diverse array of plant extracts has been reported. However, a clear understanding of the mechanism of green synthesis of NPs is lacking. Although most studies have neglected to analyze the green-synthesized NPs (GNPs) for the presence of compounds derived from the extract, several studies have demonstrated the conjugation of sugars, secondary metabolites, and proteins in these biogenic NPs. Despite several reports on the bioactivities (antimicrobial, antioxidant, cytotoxic, catalytic, etc.) of GNPs, only a handful of studies have compared these activities with their chemically synthesized counterparts. These comparisons have demonstrated that GNPs possess better bioactivities than NPs synthesized by other methods, which might be attributed to the presence of plant-derived compounds in these NPs. The ability of NPs to bind with organic compounds to form a stable complex has huge potential in the harvesting of precious molecules and for drug discovery, if harnessed meticulously. A thorough understanding of the mechanisms of green synthesis and high-throughput screening of stabilizing/capping agents on the physico-chemical properties of GNPs is warranted to realize the full potential of green nanotechnology.

## 1. Introduction

**Figure 1:**
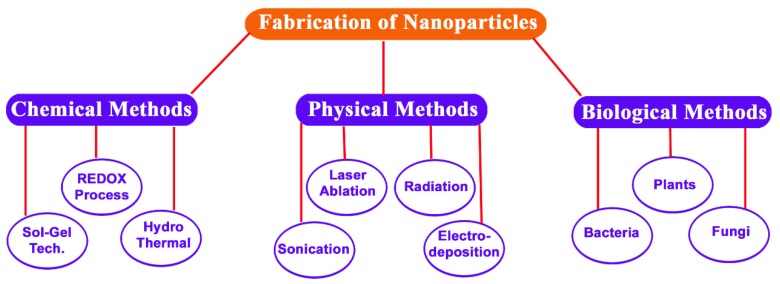
Schematic presentation of various methods used in the synthesis of metal NPs.

Nanoparticles (NPs) are particles that range 1–100 nm in size. Owing to the large surface area to volume ratio, NPs exhibit enhanced catalytic reactivity, biological activities, thermal conductivity, non-linear optical performance, and chemical steadiness in comparison to their bulk form. As a result of these properties, NPs are used in health, food, feed, space, chemical, cosmetic industries, and in agriculture [1,2,3]. NPs can be synthesized by several methods, including physical and chemical processes (Figure 1). However, as a result of the difficulties in scaling-up the physical processes and the usage of toxic synthetic chemicals that could be carried over by the NPs in chemical processes, alternative methods, such as green synthesis, have been developed. Although green chemistry methods have been practiced in agriculture, consumer products, and medicines for many years, the application of green chemistry to synthesize nanoparticles (NPs) is relatively recent.

Green synthesis is based on redox reaction, in which metal ions are reduced to stable NPs by the components of an organism or its extract. Although living organisms, such as algae, fungi, bacteria and plants, could synthesize NPs in vivo [4,5,6,7,8,9,10,11,12], plant extract-mediated, in vitro green synthesis of NPs has gained popularity due to its simplicity, low cost, eco-friendly nature, and easiness to scale-up [13,14]. Although plant extract-mediated green synthesis is a recent technology, a contrasting process has been used for the preparation of Bhasma (ash of metal) in Ayurveda, a traditional Indian medicine practiced for more than 2000 years. In the former, metal ions are reduced and stabilized as NPs by the components of the extract (bottom-up approach); by contrast, in the latter, NPs are produced by the calcination of metal sheets (top-down approach), which are macerated with medicinal plant extracts to obtain therapeutic potential.

Currently, the number of reports on green-synthesized nanoparticles (GNPs) is increasing exponentially. Extracts derived from diverse plant species, their organs, and isolated compounds are being successfully used in the green synthesis of NPs. In addition to being eco-friendly, NPs can be synthesized using agricultural and industrial waste to make the process more sustainable [15,16]. GNPs often possess better bioactivities [17,18,19,20] and catalytic characteristics compared to their counterparts, which are synthesized by other methods [21,22]; this is probably a result of the compounds attached to their surface [14]. The ability of plant secondary metabolites to bind or conjugate with NPs upon green synthesis could be exploited for the purification of compounds and drug discovery. Although the widespread occurrence of phenolic compounds in the plant kingdom could explain the ability of plant extracts to reduce metal ions, the mechanism of green synthesis is not fully understood. In this article, the current status and potential applications of the green synthesis of NPs with special emphasize on secondary metabolites participating in this process are discussed.

## 2. Mechanism of Green Synthesis of NPs by Plants

A process known as bioaccumulation, which provides plants with the ability to detoxify metal ions, can explain the mechanism of NP synthesis by plants. When plants absorb metal ions at a rate faster than that of their removal by catabolism, the excess metal ions accumulate in the plant tissues. The presence of metals at toxic levels can induce excessive reactive oxygen species (ROS) production in the cells and damage cellular macromolecules resulting in serious morphological, metabolic, and physiological irregularities in plants. To counteract the metal toxicity, plants are equipped with sophisticated chelation mechanisms to detoxify metals [23]. Cysteine-rich oligopeptides, phytochelatins, and low molecular weight proteins named metallothioneins can form complexes with metals and eventually remove the metal ions via vacuolar sequestration [24].

To maintain the ROS homeostasis upon metal toxicity, cellular enzymatic antioxidant systems are activated [25]. In addition to the enzymatic antioxidant mechanisms, plant secondary metabolites, such as phenolic compounds, can assist the cells in the maintenance of ROS homeostasis [26,27]. Induction of plant secondary metabolism in response to metal stress has been frequently reported. Stimulation of key enzymes of the phenylpropanoid pathway, such as phenylalanine ammonia-lyase (PAL) and chalcone synthase (CHS), has been observed in plants upon exposure to Cu, Cd, Al, Pb, and Ni [28]. Pteris vittata, a hyperaccumulator, produces high concentrations of chlorogenic acid derivatives and a-type procyanidin upon Cu stress [29]. Similarly, elevation of phenolic content in maize exposed to Al [30] and Phaseolus vulgaris exposed to Cu [31] has been reported. The enhanced biosynthesis of phenolic compounds by the plants under metal stress might be due to the high tendency of these compounds to chelate metals [32]. Several flavonoids are capable of chelating metal ions by forming stable complex through their multiple hydroxyl (–OH) groups and the carbonyl moiety. For instance, quercetin, a flavonoid that possesses three potential bidentate binding sites, namely α-hydroxy-carbonyl, β-hydroxy-carbonyl or catechol having two –OH groups in ortho positions, can form stable complex with metal cations such as Mo(VI), Fe(II)/Fe(III), Cu(II), Zn(II), Al(III), Tb(III), Pb(II), Co(II) by quercetin has also been reported [33].

**Figure 2:**
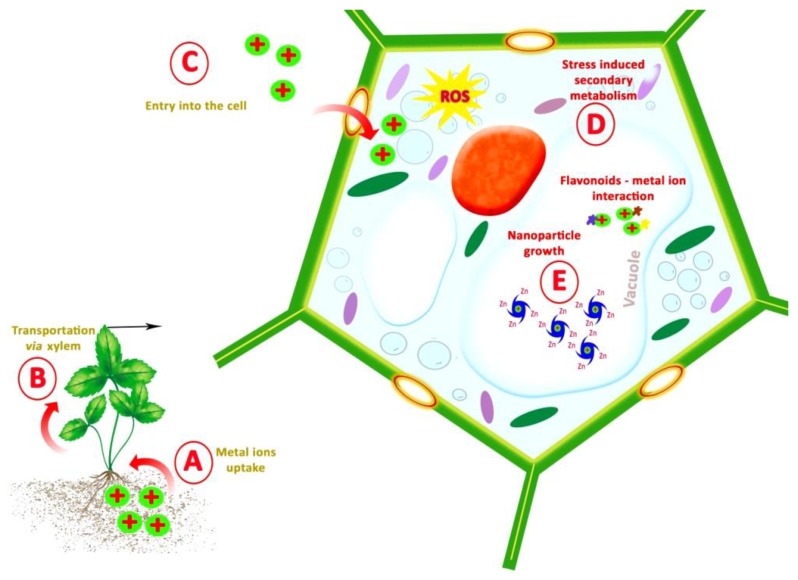
A scheme describing the possible mechanism of in vivo green synthesis of NPs.

When serving as antioxidants in a concentration-dependent manner to scavenge ROS, phenolic compounds are toxic to the cell due to their high chemical reactivity and protein-denaturing properties [34]. Hence, plants transfer these compounds to compartments with lower biosynthetic activity, such as the extracellular space and the vacuole. Flavonoids are synthesized in the cytosol and transported into the vacuole for storage [35]. The multidrug and toxic efflux transporter TT12, which is localized in the vacuolar membrane, mediates flavonoid transport by an H+-antiport mechanism into the vacuole [36]. Thus, the accumulation of metal ions and antioxidant phenolic compounds in the same cellular compartment might facilitate the formation of NPs (Figure 2).

Many plant species have the ability to synthesize and store NPs in their cells. For example, *Medicago sativa* (alfalfa) plants grown on an AuCl_4_ rich environment accumulated 4 nm size Au NPs in their tissues [7]. Similarly, when grown on an Ag ion rich medium, *M. sativa* accumulated Ag NPs [8]. *M. sativa* plants could accumulate Ag NPs of 50 nm size to about 13.6% of their fresh weight when grown on a solution containing AgNO_3_ [9]. Under the same conditions, *Brassica juncea* accumulated similar NPs to about 12.4% of fresh weight [9]. The uptake of AgNO_3_, sodium dithiosulfatoargentate [Na_3_Ag(S_2_O_3_)_2_], and diamine silver nitrate [Ag(NH_3_)_2_NO_3_] by hydroponically grown *B. juncea* and the conversion of these salts to silver metal NPs has been quantified [10]. *Brassica napus*, which was cultured on medium containing AgNO_3_, accumulated Ag NPs in the regenerated leaves [11]. Spatial distribution of Ag NPs in the chloroplast, cytoplasmic spaces, vacuolar, and nucleolar regions was confirmed by transmission electron microscopic analysis of tissues obtained from *Vigna radiata* plants exposed to AgNO_3_ [12]. Interestingly, the ability of *Phragmites australis* and *Iris pseudacorus* to transform Cu ions into Cu NPs in and around their roots has been revealed when grown in Cu-contaminated soil in the natural environment [37].

## 3. Mechanism of Green Synthesis of NPs by Plant Extracts

**Figure 3:**
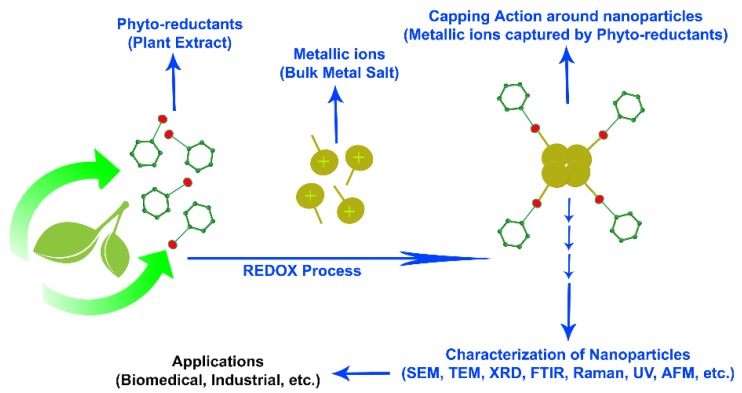
A scheme describing the mechanism of in vitro green synthesis of nanoparticles.

A similar mechanism as discussed above could be operating during green synthesis of NPs by plant extracts in vitro. Figure 3 schematically describes the formation of metallic NPs from the corresponding metal ions. When metallic salt dissociates into cation and anion, cations will be saturated to form hydroxyl complexes. Immediately after the supersaturation of hydroxyl complexes, crystillite growth of metal with oxygen species starts to originate. This results in the formation of crystalline planes with different energy levels. Heat plays a key role in providing energy to the reaction system. The process continues until activation of the capping agent from the plant extracts, which will ultimately arrest the growth of high-energy atomic growth planes. This results in the formation of specific type NPs. Generally, during the synthesis, the reducing agents donate electrons to the metal ions and convert them to NPs. These NPs exist at a high-surface energy state and tend to convert to their low-surface energy conformations by aggregating against each other. Thus, the presence of higher amounts of reducing agents and stabilizing agents prevents the aggregation of nanoparticles and promotes production of smaller NPs. Additionally, proteins can trap metal ions on their surface and convert them to their corresponding nuclei, which could further aggregate and, consequently, form NPs [38]. Amine groups of proteins, hydroxyl and carboxyl groups of polyphenols and amino acids, hydroxyl groups of polysaccharides, and carboxyl groups of organic acids chelate metal ions and suppress the superoxide-driven Fenton reaction (which is believed to be the most important source of ROS), catalyzing the formation of metallic NPs.

Although it is essential to form a protein-metal ion complex for the vacuolar sequestration of metal ions during in vivo accumulation of NPs, the role of proteins upon in vitro green synthesis is not clear. Interestingly, plant extracts possess the capacity to reduce metal ions and produce NPs even after boiling [39,40,41,42]. Although boiling could denature proteins by altering their secondary and tertiary structures, the peptide bonds of the primary structure between the amino acids are left intact. Because all structural levels of the protein determine its function, the denatured protein can no longer be fully functional. It has been stated that the protein can bind to Au NPs, either through free amino groups or cysteine residues; the surface-bound protein lead to the stabilization of the NPs [43].

## 4. Secondary Metabolites in Plant Extract-Mediated Green Synthesis of NPs

Synthesis of NPs using plant extracts has been reported in several plant species (Table 1). A wide range of molecules, ranging from proteins to various low molecular weight compounds such as terpenoids, alkaloids, amino acids, alcoholic compounds, polyphenols (catechin, flavones, taxifolin, procyanidins of various chain lengths formed by catechin and epicatechin units, and phenolic acids), glutathiones, polysaccharides, antioxidants, organic acids (ascorbic, oxalic, malic, tartaric, and protocatechuic acid), quinones etc., have been reported to play a role in the green synthesis of NPs. The participation of sugars, terpenoids, polyphenols, alkaloids, phenolic acids, and proteins in the reduction of metal ions into NPs and in supporting their subsequent stability has also been postulated [38]. As seen in Table 1, flavonoids have been the compounds most commonly reported/ predicted to participate in the green synthesis.

Although researchers face significant challenges in identifying the elements participating in the green synthesis of NPs, Fourier-transform infrared spectroscopy (FT-IR) analyses have been used to obtain clues on the biomolecules possibly involved in the reduction of the metal ions and capping. A FT-IR analysis of Ag NPs synthesized using *Acacia mearnsii* bark extract suggested that compounds with hydroxyl, alkyne, carboxyl, and amide groups of the monoterpenoids, sesquiterpenes, and phytols might have participated in these processes [140]. Similar analysis of Au NPs green-synthesized using *Suaeda monoica* leaf extract indicated that the biomolecules with carboxyl, amine, and hydroxyl functional groups were involved in the reduction of Au ions [91]. Isolated flavonoids, terpenoids, chlorogenic acid, etc. have been successfully used in the green synthesis of NPs. Highly monodispersed (18.24 nm) spherical Au NPs could be synthesized using kaempferol as the in situ reductant and stabilizer [141]. The ability of terpenoid fractions isolated from *Andrographis paniculata* leaves for the green synthesis of ZnO NPs has also been demonstrated and confirmed by the presence of C=O functional group in the NPs [142]. Au NPs were synthesized using chlorogenic acid as reductant, and the corresponding FT-IR spectra indicated that an –OH functional group was likely involved in the synthesis [143].

## 5. Flavonoids are the Major Contributors of Green Synthesis of NPs

High performance liquid chromatography (HPLC) analysis of Ag NPs green synthesized using *Withania somnifera* leaf extract revealed that several phenolic compounds present in the extract were selectively trapped in the Ag NPs [14]. On the basis of their characteristic UV-vis spectra and further confirmation by co-elution with pure standards, these compounds were identified as catechin, p-coumaric acid, and luteolin-7-glucoside. In addition, a major compound that appeared in the NPs was not found in the original extract, which might be a derivative resulting from the interaction of some withanolide derivatives with Ag ions.

Jain and Mehata [58] were able to green synthesize Ag NPs using both leaf extract of *Ocimum sanctum* and a flavonoid (quercetin) present in the extract separately. Their results revealed that both Ag NPs showed similar optical, morphological, and antibacterial characteristics, demonstrating that quercetin was responsible for the NP synthesis. Other flavonoids such as hesperidin, naringin, and diosmin have also been shown to be involved in the green synthesis of Ag NPs, with the size and shape distribution varied between the compounds. These authors concluded that the –OH group was involved in the reduction of Ag ions into Ag NPs [144].

Flavonoids are a family of natural polyphenolic compounds that include flavone, flavonol, flavanone, flavanonol, and isoflavone derivatives. The skeleton of flavonoids consists of two phenyl rings (A and B), connected by an oxygenated heterocycle ring C, and is hydroxylated in several positions. These compounds have important roles in plants because they participate in the response to biotic and abiotic stresses [145]. Much attention has been paid to the chelating properties and antioxidative activities of natural flavonoids, as they are important for plant physiology and desirable for human health. A flavonoid-metal complex may be a target for ROS and free radicals. However, this complex can be a catalytic center for Fenton reaction and the ligand moiety may act as an acceptor for hydroxyl radical. It has been reported that the antioxidant activity of flavonoid-metal complexes is higher than that of free ligands [146,147].

**Figure 4:**
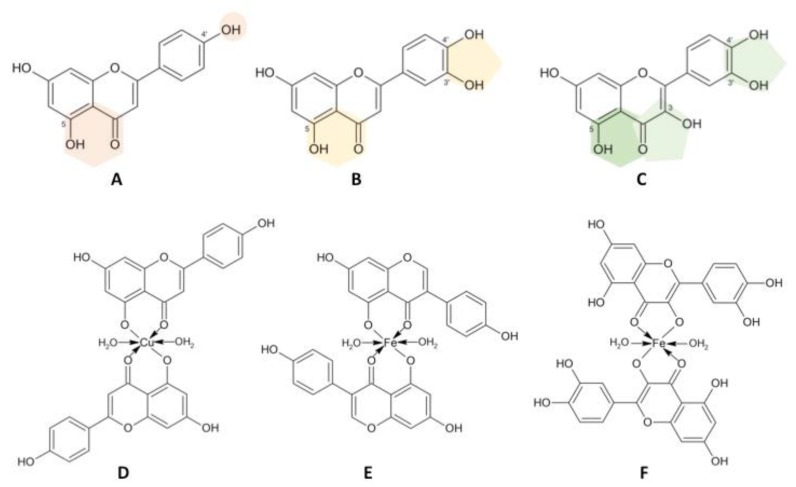
Metal-chelating properties of flavonoids.

The number of hydroxyl groups and the structure of flavonoids are important for metal-binding activity (Figure 4). Simple aglycones, such as chrysin, apigenin and genistein, may accept metal ion in one coordination pocket between the 4-carbonyl group and 5-hydroxyl group. Divalent metal ions Cu^2+^, Co^2+^, and Ni^2+^ are bound by two chrysin ligands [148]. Moreover, genistein and biochanin a chelate Cu^2+^ and Fe^3+^ with a 1:2 (metal:ligand) stoichiometry. Complexes of isoflavones with Cu have higher antioxidant potential than the free ligands, as revealed via 2,2-diphenyl-1-picrylhydrazyl (DPPH) reduction assays. Fe chelates were shown to have prooxidant potential [149]. In another report, apigenin was found to bind Al^3+^ and Fe^2+^ with a 1:3 (metal:ligand) stoichiometry in a dioxan water solution. In this case, the 4′-hydroxyl group was the coordination site [150]. Apigenin, naringenin, and hesperitin can chelate Cu ions and create coordination pocket between 4-carbonyl and 5-hydroxyl groups. Such complexes have a strong DNA-binding properties and cytotoxicity [151].

Bicalein is a highly bioactive flavonoid that is characterized by three possible bidentate binding sites (4-carbonyl and three hydroxyl groups at carbons number 5, 6, 7). In this case, metal:bicalein complexes can be created by using only one binding site. Either hydroxyls at 5- and 6- carbon atoms or 6-hydroxyl-7-hydroxyl are potentially the binding sites of Fe^2+^ and Fe^3+^ ions, with a 1:1 and 1:2 stoichiometry. The Fe-bicalein complexes have high antioxidant properties due to inhibition of the Fenton reaction [152,153]. Similarly, two potential bidentate binding sites are present in the luteolin structure (5-hydroxyl-4-carbonyl and 3′4′-hydroxyl groups of the ring B) and both sites can bind Al^3+^ ions in the molar ratio 2:1 (metal:ligand). The separation of the coordination pocket is important for increasing the chelating properties [154]. Quercetin has three potential bidendate binding places. The stable complexes of quercetin were reported for a large number of metal ions, such as Fe^2+^, Fe^3+^, Cu^2+^, Zn^2+^, Co^2+^, Pb^2+^, Al^3+^ [155]. 3-hydroxyl and 4-carbonyl groups of quercetin can chelate Fe^2+^ with a 1:2 (metal:ligand) stoichiometry. Quercetin-iron complex is characterised by high free radical scavenging, DNA binding and antibacterial activities [156].

Cherrak, et al. [33] reported on the chelating properties of quercetin, O-methylated quercetins, rutin, and catechin. The most stable complexes with Fe^3+^, Zn^2+^, and Cu^2+^ were observed for catechin, quercetin, and rutin compared to O-methylated analogues. Moreover, spectrophotometric studies of O-methylated quercetins showed the binding sequence with the iron ion: 3′4′-OH > 3-OH >> 5-OH [33]. In summary, the modification of free hydroxyl groups in the flavonoids structure causes changes to both chelating and antioxidant properties. O-methylated flavonoids are weaker chelators than the free ligands. The aglycones have stronger antioxidant activities in comparison to the glycosides, e.g., baicalein-baicalin or luteolin-luteolin-4′-O-glucoside [147].

## 6. Green-Synthesized NPs are Highly Bioactive and Biocompatible

As discussed above, GNPs are often capped with secondary metabolites [14]. In addition to providing stability to the GNPs as capping agents or stabilizers, the presence of these compounds might enhance the bioactivities of these NPs (Table 2). The scavenging activities of Ag NPs prepared using three different natural polyphenols, epigallocatechin-3-gallate (EGCG), resveratrol (RSV), and fisetin, were highly correlated with their secondary metabolite content [157]. Green-synthesized Ag NPs using white rot fungi *Pycnoporus* broth showed better antimicrobial activity against the pathogenic bacteria in comparison to the chemically synthesized ones [158]. Microbiological tests performed using varying concentrations of green (aloe extract) and chemical ZnO NPs showed that green ZnO nanoparticles had enhanced biocidal activity against various pathogens compared to the chemical ZnO NPs [17]. It was observed that the green-synthesized Ag NPs using *Salvadora persica* root extract exhibited comparable or better antibacterial activities than the chemically obtained Ag NPs [18]. A bioactivity comparison of Ni NPs prepared via the chemical and green routes (*Desmodium gangeticum* aqueous root extract) suggested that NPs prepared by the green route had better antioxidant and antibacterial activity, without any toxicity towards epithelial cell line and Wistar rats [19].

Interestingly, green-synthesized ZnO NPs, which had been stabilized by plant metabolites, varied in their anti-diabetic activity based on their size in streptozotocin (STZ)-induced diabetic mice [20]. Au NPs synthesized using methanolic extract of *Azolla microphylla* showed excellent antioxidant activity [159]. Au NPs produced from *Hypoxis hemerocallidea* exhibited antibacterial activity against *Staphylococcus aureus, Staphylococcus epidermidis*, *Escherichia coli*, and *Pseudomonas aeruginosa*, whereas Au NPs produced from *Galenia africana* only exhibited antibacterial activity against *P. aeruginosa* [94]. The Fe_3_O_4_ NPs synthesized using agro-waste extracts exhibited higher removal (>90%) of antibiotics than Fe_3_O_4_ NPs synthesized by a conventional method [15]. The Cd NPs synthesized using marigold petal extracts showed better larvicidal activity against mosquito larvae compared to the Cd NPs synthesized using rose petal extracts [120]. An *Azadirachta indica* extract-mediated reduction of Ag ions resulted in the formation of different sizes of NPs (4.74 nm, 8.17 nm, 14.23 nm, and 18.98) when the aqueous extract was not boiled [160], whereas the NPs prepared using boiled extract were of an average size of 34 nm [46]. Moreover, although the latter showed antibacterial activities against both gram positive and gram-negative bacteria, the former did not show activity against gram-positive bacteria.

## 7. Potential Applications of Green-Synthesized NPs

In addition to the several bioactivities listed above, GNPs also have been found to possess several industrial applications due to the presence of plant compounds (Table 3). For instance, enzymes are important biocatalysts in modern biotechnology but are highly unstable in nature. The thermal, pH, and storage stability of α-amylase could be improved by immobilization with naringin-functionalized magnetite nanoparticles [210]. Ag NPs synthesized using *O. sanctum* and *Chenopodium aristatum* showed good catalytic activity in degradation of 4-nitrophenol [21,22]. Similarly, Ag NPs synthesized using *Thuja occidentalis* extract could be used as a soil conditioner and plant growth promoter [211]. Cu NPs synthesized using *Lawsonia inermis* extract could be used to prepare electrical-conducting nanobiocomposites [123]. CuO NPs green-synthesized using *Ocimum tenuiflorum* extract could be used as non-enzymatic glucose biosensor [212].

The ability of plant secondary metabolites to chelate metal ions in the production of stable complexes and their potential to conjugate with NPs has opened a new window for NP use in harvesting these natural products. The ability to form a NP-secondary metabolite complex upon green synthesis provides us an opportunity to establish a relationship between NP type and compound classes, which would have impact on the nanoharvesting of compounds from live plants or tissues. During nanoharvesting, the metabolites are adsorbed onto the NPs and extrudes from plant cells to the medium and the metabolites can be separated through elution and magnetization [217]. Nanoharvesting eliminates the use of organic solvents, allows for the spectral identification of the isolated compounds, and provides new avenues for the use of nanomaterials for coupled isolation and testing of bioactive properties of plant-synthesized compounds.

**Figure 5:**
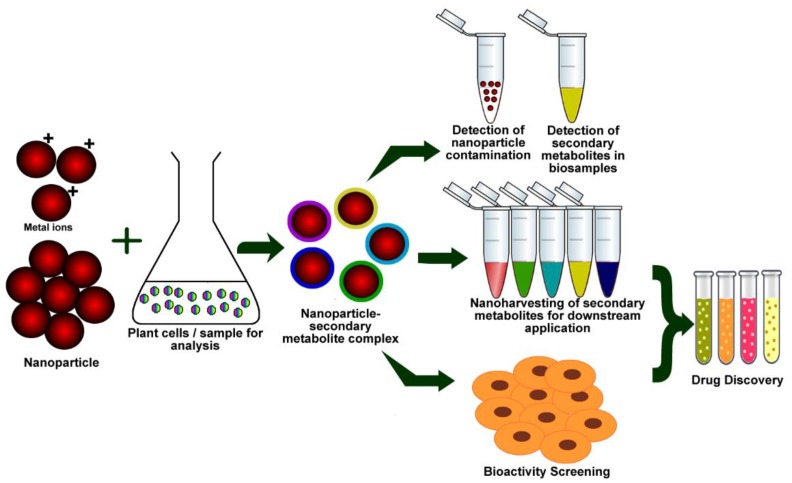
A scheme showing possible nanotrapping/nanoharvesting and drug discovery applications of green synthesis.

Kurepa, et al. [213] showed that TiO_2_ NPs enter *Arabidopsis thaliana* plant cells, conjugate enediol and catechol group-rich flavonoids in situ, and exit plant cells as flavonoid-nanoparticle conjugates. The compound adsorption capacities of NPs could be further improved by functionalization [217]. For instance, adsorption capacity of SiO_2_ NPs towards quercetin was higher upon TiO_2_ functionalization in comparison to non-functionalized and decyl group functionalized SiO_2_ NPs due to possible binding of quercetin to the metal oxide [208]. This adsorption capacity increased linearly with surface coverage of TiO_2_, emphasizing the correlation between functional surface and quercetin adsorption. Similar to in vivo nanotrapping, in vitro green synthesis of NPs using plant extracts can be extended further to develop high throughput tools to extract specific classes of compounds from crude extracts (Figure 5).

This NPs-secondary metabolite conjugation property can also be used in different fields of industrial biotechnology. Nanoparticle-mediated delivery of medicinally important flavonoids and other biomolecules will increase their therapeutic efficacy. As a potential drug delivery system, hesperitin-conjugated gold nanoparticles enhanced the treatment of hepatocellular carcinoma by minimizing the side effects and reduced the dose of chemotherapy drug [222]. Recently, several reports have addressed the potential risk of NPs to both human health and the environment, which necessitates a method to detect them in food and other samples. A green, facile, and rapid method using a flavonoid-assisted method was standardized to extract and detect the TiO_2_ NPs in food samples [214]. It was also applicable in identifying flavonoid traces in biological samples, such as urine and blood [215].

## 8. Conclusions

Although the ability of extracts from diverse plant species to synthesize NPs could be explained by the widespread occurrence of polyphenolic compounds in the plant kingdom, a precise understanding of the green synthesis process is needed to realize the full potential of this process in medical and industrial applications. In spite of the facile synthesis of NPs via a green method, obtaining homogeneously dispersed NPs is a huge challenge, as several parameters including temperature, pH of the system, nature of the capping agent, concentration of active compounds, etc. might play vital roles in defining the size and morphology [225]. Au NPs synthesized using *Cinnamomum zeylanicum* leaf broth differed in their shape, which was based on the concentration of the extract [39]. At lower concentrations of the extract, the formation of prism-shaped NPs dominated and, at higher concentrations however, mostly spherical NPs were formed [39]. Hence, reducing, capping, or stabilizing agents participating in the green synthesis need to be further analyzed to specify the NP structural relationship.

A high-throughput analysis of plant extracts with diverse metal ions would provide clues as to whether there is any correlation between the specific compounds and the NPs generated. This would have a huge potential in the trapping of compounds and proteins using metal ions or NPs, which are difficult or expensive to purify by other means. NPs green-synthesized using medicinal plant extracts should be tested for various bioactivities in comparison with their chemically or physically synthesized counterparts to understand whether the bioactivities observed could be attributed to the presence of capping agents in the NPs. Considering that NPs bioactivities also differ between their size shape and zeta potential, similar NPs needs to be compared. For example, in spite of excellent antibacterial activities reported against antibiotic resistant strains, it is not clear whether this is a result of the NPs, the compounds attached to them, both, or, conversely, a result of the other compounds present in the extract; such comparisons require further study.

In the light of above discussion, it has been extensively reported that GNPs surface chemistry is helpful in the advancement of biomedical applications. However, most studies have shown that GNPs are subjected to calcinations using a blast furnace at ultra high temperatures [225,226,227] to attain the highest level of crystalline morphology before they can be used. This will surely provide a pure-phase crystalline structure, but the surface-attached phytochemicals as capping agents will be decomposed. Accordingly, to achieve GNPs with functionalized surfaces and sticky capping agents for advanced biological applications, we must improve the synthesis modes.

## Figures and Tables

**Table 1:** Plant components possibly involved in the green synthesis of nanoparticles from various plant species.

Plant Species	NPs	Metabolites Identified in the Extract/NPs	Reference
*Coleus aromaticus*	Ag	Flavonoids	[44]
*Syzygium cumini*	Ag	Flavonoids	[45]
*Azadirachta indica*	Ag	Flavanoids, terpenoids	[46]
*Citrus sinensis*	Ag	Flavonoids, ascorbic acid, volatile oils	[47]
*Zingiber officinale*	Ag	Flavonoid, alkaloids	[48]
*Ocimum sanctum*	Ag	Flavonoid, proteins, gallic acid, terpenoids	[49]
*Glycyrrhiza glabra*	Ag	Flavonoids, thiamine and terpenoids	[50]
*Nigella arvensis*	Ag	Flavonoids, alkaloids	[51]
*Dioscorea bulbifera*	Ag	Flavonoids, polyphenols	[52]
*Lantana camara*	Ag	Flavonoids, glycosides and carbohydrates	[53]
*Helianthus annuus*	Ag	Flavonoids, proteins, amino acids, amides terpenoids	[54]
*Rosmarinus officinalis*	Ag	Polyphenols	[55]
*Mimusops elengi*	Ag	Polyphenols	[56]
*Syzygium cumini*	Ag	Polyphenols	[57]
*Ocimum sanctum*	Ag	Quercetin	[58]
*Acalypha indica*	Ag	Quercetin, plant pigment	[59]
*Gardenia jasminoides*	Ag	Rutin, gallic acid, chlorogenic acid	[60]
*Withania somnifera*	Ag	Catechin p-coumaric acid, luteolin-7-glucoside, withanolides	[14]
*Lippia citriodora*	Ag	Verbascoside, isoverbascoside, chrysoeriol-7-O-diglucoronide, luteonin-7-O-diglucoronide	[61]
*Decalepis hamiltonii*	Ag	Polyols, phenols	[62]
*Achyranthes aspera*	Ag	Polyols	[63]
*Hybanthus enneaspermus*	Ag	Several bioactive phytochemical compounds	[64]
*Desmodium triflorum*	Ag	Ascorbic acid	[65]
*Sesuvium portulacastrum*	Ag	Flavones, proteins, terpenoids	[66]
*Solanum xanthocarpum*	Ag	Alkaloids, phenolics, sugars	[67]
*Mentha piperita*	Ag	Alkaloids, flavones, steroids, polysaccharides, amino acids, oximes, proteins, menthol	[41]
*Anacardium occidentale*	Ag	Proteins, polyols	[68]
*Dioscorea bulbifera*	Ag	Diosgenin, ascorbic acid	[52]
*Iresine herbstii*	Ag	Phenolic compound	[69]
*Trianthema decandra*	Ag	Catechins, hydroxyflavones	[70]
*Morinda pubescens*	Ag	Catechins, hydroxyflavones	[71]
*Carica papaya*	Ag	Catechnis, hydroxyflavones	[72]
*Annona squamosa*	Ag	Alkaloids, glycoside, saponins, tannins, phenolic, carbohydrates	[73]
*Trianthema decandra*	Ag	Saponin	[74]
*Aegle marmelos*	Ag	Tannin	[75]
*Rosa rugosa*	Ag	Carboxylate, amine groups	[76]
*Hibiscus rosa- sinensis*	Ag	Carboxylate ion groups	[77]
*Leonuri herba*	Ag	Hydroxyl, polyphenols groups	[78]
*Lonicera japonica*	Ag	Phenolic and hydroxyl groups of chlorogenic acid	[79]
*Mangifera indica*	Ag	Ketone, aldehydes, hydroxyl, carboxyl groups	[80]
*Eucalyptus*	Ag	Alcohol, phenols, alkylaldehyde	[81]
*Alternanthera sessilis*	Ag	Tannins, carbohydrates, proteins, ascorbic acid	[82]
*Boswellia serrata*	Ag	Proteins	[83]
*Piper betle*	Ag	Proteins	[84]
*Plumeria rubra*	Ag	Proteins	[85]
*Jatropha curcas*	Ag	Cyclic peptides (curcacycline A and curcacycline B)	[86]
*Hibiscus rosa- sinensis*	Au	Flavonoids	[87]
*Vitis vinifera*	Au	Flavonoids	[88]
*Mangifera indica*	Au	Favonoids, terpenoids, thiamine	[89]
*Abutilon indicum*	Au	Flavonoids, phenolic compounds	[90]
*Suaeda monoica*	Au	Flavonoids, terpenoids, soluble proteins	[91]
*Sesbania grandiflora*	Au	Flavonoids, polyphenols	[92]
*Citrus maxima*	Au	Flavonoids, terpenes, vitamins	[93]
*Hypoxis hemerocallidea*	Au	Flavonoids, terpenoids, phenolic compounds and/or carbohydrates	[94]
*Galenia africana*	Au	Flavonoids, terpenoids, phenolic compounds and/or carbohydrates	[94]
*Nigella arvensis*	Au	Flavonoids, phenolic compounds	[95]
*Butea monosperma*	Au	Polyphenols	[96]
*Sterculia acuminata*	Au	Polyphenols	[97]
*Terminalia arjuna*	Au	Polyphenols	[98]
*Terminalia catappa*	Au	Hydroxyl group of phenols	[99]
*Hygrophila spinosa*	Au	Hydroxyl group	[100]
*Cassia auriculata*	Au	Hydroxyl group	[101]
*Platycodon grandiflorum*	Au	Hydroxyl group	[102]
*Phoenix dactylifera*	Au	Hydroxyl group	[103]
*Lansium domesticum*	Au	Carboxylic acid	[104]
*Salix alba*	Au	Proteins, metabolites having functional groups of amines, alcohols, ketones, aldehydes, carboxylic acids (salicin)	[105]
*Cinnamomum zeylanicum*	Au	Proteins	[39]
*Ficus benghalensis*	Au	Proteins	[106]
*Jatropha*	Au	Proteins	[16]
*Morinda citrifolia*	Au	Proteins	[42]
*Gymnema sylvestre*	Au	Proteins, polypeptides	[107]
*Olea europaea*	Au	Proteins	[40]
*Trianthema decandra*	Au	Saponin	[74]
*Terminalia arjuna*	Au	Hydrolyzable tannins	[108]
*Elaeis guineensis*	Au	Phenolic, carboxylic, amines	[109]
*Mentha piperita*	Au	Menthol	[41]
*Argemone mexicana*	Au	Phosphorous compounds	[110]
*Tamarindus indica*	Au	Phenolic compounds	[111]
*Averrhoa bilimbi*	Au	Phenols, tertiary amides	[112]
*Couroupita guianensis*	Au	Phenol group	[113]
*Syzygium jambos*	Au	Saccharides, phenolics	[114]
*Zostera noltii*	Au	Flavone sulfates	[115]
*Ipomoea carnea*	Au	Polysaccharides, protein	[116]
*Mirabilis jalapa*	Au	Polysaccharides	[117]
*Panax ginseng*	Au	Polysaccharides, phenolic compounds	[118]
*Galaxaura elongata*	Au	Glutamic acid, hexadecanoic acid, oleic acid, 11-eicosenoic acid, stearic acid, gallic acid, epigallocatechin, catechin, epicatechin gallate	[119]
*Tagetes* sp. and *Rosa* sp.	Cd	Alcoholic, amide, C–C, –OCH3 groups (tannins, flavonoids, alkaloids and carotenoids)	[120]
*Punica granatum*	Cu	Flavonoids, alkaloids, polyphenols	[121]
*Cymbopogon citratus*	Cu	Polyphenols, proteins	[122]
*Lawsonia inermis*	Cu	Phenolic compounds	[123]
*Euphorbia granulate*	Pd	Hydroxyflavones, phenolics	[124]
*Hippophae rhamnoides*	Pd	Flavonoids	[125]
*Delonix regia*	Pd	Polyphenols	[126]
*Cacumen platycladi*	Pt	Flavonoids, proteins	[127]
*Diospyros kaki*	Pt	Terpenoids	[128]
*Dioscorea bulbifera*	Pt-Pd	Hydroxyl group of polyphenolic compounds	[129]
*Cassia fistula*	ZnO	Flavonoids, polyphenols	[130]
*Azadirachta indica*	ZnO	Flavonoids, phenolic acid, terpenoids, protein	[131]
*Rosa canina*	ZnO	Phenolic and carboxylic acids	[132]
*Aloe barbadensis*	ZnO	Phenol, amines, alcohol groups	[133]
*Agathosma betulina*	ZnO	Hydroxyl group	[134]
*Trifolium pratense*	ZnO	Hydroxyl group	[135]
*Parthenium hysterophorus*	ZnO	Phosphorus compound, secondary sulfonamide, monosubstituted alkyne	[136]
*Anisochilus carnosus*	ZnO	Phenol, carboxylic acid	[137]
*Coptis chinensis*	ZnO	Alcohol, carboxylic acid, alkyl halide, alkynes	[138]
*Calotropis procera*	ZnO	Hydroxyl groups, aldehydes, amines, ketones, carboxylic acids	[139]

**Table 2:** Bioactivities of green-synthesized NPs.

NPs	Plant Species Used	Bioactivity Reported	Reference
Ag	*Withania somnifera*	Antibacterial, anticandidal	[14]
Ag	*Capsicum frutescens*	Antibacterial	[104]
Ag	*Crocus sativus*	Antibacterial	[161]
Ag	*Datura stramonium*	Antibacterial	[162]
Ag	*Prosopis glandulosa*	Antibacterial	[163]
Ag	*Azadirachta indica*	Antibacterial	[46]
Au	*Hypoxis hemerocallidea*	Antibacterial	[94]
Au	*Galenia africana*	Antibacterial	[94]
Cu	*Terminalia catappa*	Antibacterial	[164]
Se	*Azadirachta indica*	Antibacterial	[165]
Pt	*Taraxacum laevigatum*	Antibacterial	[166]
TiO_2_	*Trigonella foenum-graecum*	Antibacterial	[167]
Ag_2_O	*Ficus benghalensis*	Antibacterial	[168]
Ag	*Pteris tripartita*	Antibacterial, antifungal, antioxidant, antiinflammatory	[169]
Ag	*Phyllanthus amarus*	Antibacterial	[170]
Ag	*Aloe arborescens*	Antibacterial	[171]
Ag	*Syngonium podophyllum*	Anticandidal	[172]
Ag	*Euphorbia prostrata*	Antiplasmodial	[173]
Ag	*Ocimum sanctum*	Antibacterial	[58]
Ag	*Hybanthus enneaspermus*	Larvicidal	[64]
Ag	*Eclipta prostrata*	Larvicidal	[174]
Cd	Tagetes *sp. and* Rosa *sp.*	Larvicidal	[120]
Ag	*Holarrhena antidysenterica*	Larvicidal	[175]
Ag	*Tinospora cordifolia*	Larvicidal	[176]
Ag	*Chrysanthemum*	Larvicidal	[177]
Ag	*Delonix elata*	Wound healing	[178]
Ag	*Ficus krishnae*	Antibacterial, anticancer	[179]
Ag	*Andrographis paniculata*	Hepatocurative	[180]
Ag	*Lippia nodiflora*	Antioxidant, antibacterial, cytotoxic	[181]
Ag	*Tragia involucrata*	Antiurolithic	[182]
Ag	*Tagetes patula*	Antifungal	[183]
Au	*Vetiveria zizanioides*	Antifungal	[184]
Au	*Cannabis sativa*	Antifungal	[184]
Ag	*Rauvolfia serpentina*	Antimicrobial, larvicidal and cytotoxic	[185]
Au	*Cassia fistula*	Antihypoglycemic	[186]
Au	*Terminalia chebula*	Antifilarial	[187]
Au	*Euphorbia milii*	Antinociceptive, muscle relaxant, sedative	[188]
Ag	*Rubus glaucus*	Antioxidant	[189]
Au	*Punica Granatum*	Antioxidant	[190]
Au	*Azolla microphylla*	Antioxidant	[159]
CuO	*Morus alba*	Antioxidant	[191]
CuO	*Olea europaea*	Antioxidant	[192]
Au	*Acanthopanacis cortex*	Anti-inflammatory	[193]
Au	*Allium sativum*	Hepatoprotective	[194]
Au	*Trigonella foenum-graecum*	Catalytic	[195]
CuO	*Cissus quadrangularis*	Antifungal	[196]
CuO	*Ormocarpum cochinchinense*	Anticancer	[197]
Pt	*Punica granatum*	Cytotoxic	[198]
Pd	*Tinospora cordifolia*	Antifilarial, antimalarial	[199]
Pd	*Pelargonium graveolens*	Cytotoxic	[200]
Zn	*Cochlospermum religiosum*	Antibacterial, antimitotic	[201]
Zn	*Momordica charantia*	Acaricidal, pediculicidal, larvicidal	[202]
ZnO	*Ulva lactuca*	Insecticidal	[203]
ZnO	*Hibiscus sabdariffa*	Antibacterial, antidiabetic	[20]
ZnO	*Calotropis procera*	Photocatalytic	[139]
Ni	*Desmodium gangeticum*	Antioxidant, antibacterial	[19]
NiO	*Aegle marmelos*	Cytotoxic, antibacterial	[204]
CeO_2_	*Camellia sinensis*	Healing of liver sepsis	[205]
TiO_2_	*Parthenium hysterophorus*	Larvicidal, antibacterial, photocatalytic	[206]
Fe_3_O_4_	*Rosmarinus officinalis*	Leishmanicidal	[207]
CeO_2_	*Rubia cordifolia*	Anticancer	[208]
Se	*Clausena dentata*	Larvicidal	[209]
Au	*Salix alba*	Antifungal, antinociceptive, muscle relaxant	[105]

**Table 3:** Secondary metabolites conjugated with NPs.

NP	Source	Compound(s) Trapped/Conjugated	Reference
Ag	*Withania somnifera* leaf extract	Catechin, p-coumaric acid, and luteolin-7-glucoside and withanolides	[14]
TiO_2_	*Arabidopsis thaliana* seedlings	Flavonoids	[213]
TiO_2_	Food samples	Myricetin	[214]
Fe_3_O_4_	Urine and blood	Luteolin, quercetin, kaempferol	[215]
TiO_2_	Flavonoids	Flavonoids	[216]
TiO_2_-SiO_2_	Quercetin, rutin	Quercetin, rutin	[217]
TiO_2_-SiO_2_	Quercetin	Quercetin	[218]
Au	Baicalin	Baicalin	[219]
Au	Naringin	Naringin	[220]
Au	Quercetin	Quercetin	[221]
Au	Hesperetin	Hesperetin	[222]
Au	Quercetin	Quercetin	[223]
Fe_3_O_4_	Quercetin	Quercetin	[224]
Fe_3_O_4_	Naringin	Naringin	[210]
