# Correction: Marslin, G., et al. Secondary Metabolites in the Green Synthesis of Metallic Nanoparticles. *Materials* 2018, *11*, 940

**DOI:** 10.3390/ma12050806

**Published:** 2019-03-08

**Authors:** Gregory Marslin, Karthik Siram, Rajendran Kamalabai Selvakesavan, Dariusz Kruszka, Piotr Kachlicki, Gregory Franklin

**Affiliations:** 1 Ratnam Institute of Pharmacy and Research, Nellore 524346, India; marslingregory@gmail.com; 2 Department of Pharmaceutics, PSG College of Pharmacy, Coimbatore 641004, India; karthiksiram@gmail.com; 3 Institute of Plant Genetics of the Polish Academy of Sciences, Poznan 60479, Poland; kesavanrks@gmail.com (R.K.S.); dmkruszka@gmail.com (D.K.); pkac@igr.poznan.pl (P.K.); fgre@igr.poznan.pl (G.F.)

The authors have overlooked a few mistakes when rearranging the Table 1 and Table 2 and references at the final stages, which were carried-over to the published version of the review. Consequently the authors wish to make at this time the following corrections to the paper [1]:

Page 11, final paragraph: Citation [208] should be read as [218].

**References**

70. Geethalakshmi, R.; Sarada, D.V. Synthesis of plant-mediated silver nanoparticles using *Trianthema decandra* extract and evaluation of their antimicrobial activities. *Int. J. Eng. Sci. Technol.* **2010**, *2*, 970–975.
111. Correa, S.N.; Naranjo, A.M.; Herrera; A.P. Biosynthesis and characterization of gold nanoparticles using extracts of *Tamarindus indica* L Leaves. *J. Phys.: Conf. Ser.* **2016**, *687*, 012082.
225. Shah, M.; Fawcett, D.; Sharma, S.; Tripathy, S.K.; Poinern, G.E.J. Green synthesis of metallic nanoparticles via biological entities. *Materials* **2015**, *8*, 7278–7308.

The manuscript will be updated and the original will remain available on the article webpage. We would like to apologize for any inconvenience caused to the readers.

## Figures and Tables

**Table 1:** Plant components possibly involved in the green synthesis of nanoparticles (NPs) from various plant species.

Plant Species	NPs	Metabolites Identified in the Extract/NPs	Reference
*Ocimum sanctum*	Ag	Eugenols, linalool, terpenes	[49]
*Helianthus annuus*	Ag	Fatty acids, triglycerides, phenolics, tocopherols	[54]
*Solanum xanthocarpum*	Ag	Alcohols, phenols, carboxylic anions	[67]
*Morinda pubescens*	Ag	Flavonoids, triterpenoids, polyphenols	[71]
*Carica papaya*	Ag	Proteins, alcohols, phenolics	[72]
*Piper betle*	Ag	Amide, aromatic amine	[84]
*Eucalyptus*	Fe	Alcohol, phenols, alkylaldehyde	[81]

**Table 2:** Bioactivities of green-synthesized NPs.

NPs	Plant Species Used	Bioactivity Reported	Reference
Ag	*Lansium domesticum*	Antibacterial	[104]
